# Comparison of different bacteriological testing strategies and factors for bacteriological confirmation among pulmonary TB patients: a retrospective study in Tianjin, China, 2017–2018

**DOI:** 10.1186/s12879-020-05273-3

**Published:** 2020-07-29

**Authors:** Guoqin Zhang, Yuhua Zhang, Mingting Chen, Fan Zhang

**Affiliations:** 1 Tianjin Center for Tuberculosis Control, No. 124 Chifeng Road, Heping District, Tianjin, 300041 P. R. China; 2 Chinese Center for Disease Control and Prevention, Beijing, 102206 China

**Keywords:** Mycobacterium, Tuberculosis, Bacteriology, Logistic models, Risk factor

## Abstract

**Background:**

Bacteriological confirmation (BC) proportion among notified pulmonary TB patients in China is among the lowest in the world. This study was to understand the yield of BC using different testing strategies and patient-level factors associated with BC among pulmonary TB patients in Tianjin, China during 2017–2018.

**Methods:**

A retrospective study was conducted, enrolling pulmonary TB patients reported to National TB Information Management System (TBIMS) in Tianjin during 2017–2018. BC was defined as a positive result by any of the followings: smear microscopy, culture, or nucleic acid amplification test. Individual characteristics were compared between patients with positive and negative bacteriological results using contingency tables and χ^2^ test. Multivariable logistic regression was applied to analyze factors associated with BC, calculating adjusted odds ratios (aOR) and 95% confidence intervals (CI) (α = 0.05).

**Results:**

Of 6364 reported patients, 4181 (65.7%) were bacteriologically confirmed. Positivity proportion was 43.1% (2746/6364) for smear microscopy, 57.7% (3380/5853) for culture, 61.7% (1608/2605) for Xpert® MTB/RIF assay (Xpert) and 73.4% (1824/2484) for combination of the three. The unemployed (aOR = 1.5, 95% CI: 1.0–2.2) and farmers (aOR = 1.7, 95% CI: 1.1–2.8) compared with students; diagnosis by inpatient hospitals compared with TB clinics (aOR = 3.4, 95% CI: 2.6–4.4); having symptoms for ≥2 weeks (aOR = 1.4, 95% CI: 1.1–1.8); cough (aOR = 2.2, 95% CI: 1.8–2.8); blood sputum (aOR = 1.5, 95% CI: 1.0–2.2); cavitation on chest X-ray (aOR = 3.3, 95% CI: 2.5–4.3); bilateral lung lobes affected (aOR = 1.7, 95% CI: 1.4–2.2) were factors associated with BC.

**Conclusions:**

Combination test was an effective way to improve BC among pulmonary TB patients. Being unemployed, farmers, having prolonged symptoms, and more severe in TB condition were factors associated with BC. We recommend combination of tests to improve BC for pulmonary TB patients, especially who are in early stage of the disease or with conditions tend to be bacteriologically negative.

## Background

Tuberculosis (TB) is caused by *Mycobacterium tuberculosis (MTB)*. Despite a long history it remains a major threat to global health. In 2018, an estimation of 10.0 million people developed TB, and 1.5 million died from the disease [1]. Pulmonary TB is the most common type of TB, and the patients may produce droplet nuclei containing MTB through coughing, sneezing, spitting, speaking or singing, allowing it transmit from person to person. Testing for MTB in sputum samples is a direct way for pulmonary TB diagnosis, and patients with positive results are classified as bacteriologically confirmed patients [2]. Bacteriological confirmation (BC) helps TB diagnosis timely, determines contact investigation and allows further looking into susceptibility of drugs [2–4]. Failure to detect MTB among patients who actually bear significant bacilli can cause delay in TB diagnosis and treatment [5–7].

MTB testing, from the long-used sputum smear microscopy and solid media culture, to liquid culture and the most novel tool nucleic acid amplification assays, such as Xpert® MTB/RIF assay (Xpert), have improved rapidness or sensitivity [8, 9]. Despite the evolution of MTB test, still a number of pulmonary TB patients are clinically diagnosed in the absence of BC. In 2018, globally BC proportion among notified pulmonary TB was 55% [1]. In China this proportion was 37% in 2018, although improved from 32% in 2017, still among the lowest in the world [1]. With the second largest TB burden in the world, accounting for 9% in 2018, [1] China is facing a challenge of low BC among pulmonary TB patients. Tianjin is one of the four municipalities in China, where sputum smear, culture and Xpert were gradually accessible to all pulmonary TB patients for free during diagnosis. This study was to better understand the role of sputum smear, culture and Xpert in BC, and patient-level factors associated with it, in order to inspire interventions to improve BC for the whole country and also some other regions in the world.

## Methods

### Study design

A retrospective study using de-identified data from the National TB Information Management System (TBIMS) in Tianjin during 2017–2018.

### Study population

Pulmonary TB was diagnosed according to the China national standard of “Diagnosis for pulmonary tuberculosis”, either based on a positive bacteriological test or determination by a panel of physicians in the absence of bacteriological evidence [2, 6]. Demographic and clinic information of patients were collected by physicians in medical records, and entered to TBIMS within 24 h. Timeliness and quality of data reporting were supervised by Tianjin Center for TB Control. Data of pulmonary TB patients reported during 2017–2018 was exported from TBIMS in Excel forms, without variables containing identifiable information such as patients’ name, telephone number, address and personal ID number. Inclusion criteria: pulmonary TB patients notified in Tianjin during 2017–2018. Exclusion criteria: 1) patients not recorded initial sputum smear result; 2) patients who were ruled out of pulmonary TB later on.

### Bacteriological examinations

Presumable TB patients were required to collect three sputum specimens for smear microscopy, extra sputum specimens for *mycobacterium* culture and nucleic acid amplification assays. Sputum smear was performed using Ziehl-Neelsen staining, and culture was performed using either Mycobacterium Growth Indicator Tube 960 or Löwenstein-Jensen medium according to WHO guidelines [10]. Xpert® MTB/RIF (Cepheid, https://www.cepheid.com) was performed on sputum according to the manual instructions to improve the bacterium positivity as well as for Rifampicin resistance detection.

### Definition

A bacteriologically confirmed pulmonary TB patient is defined as a patient with any positive result shown by sputum smear microscopy, culture or a WHO-approved nucleic acid amplification test, such as Xpert; a patient diagnosed in the absence of the above-mentioned evidence is defined as a clinically diagnosed pulmonary TB patient.

### Data analysis

Separate positivity as well as the combined positivity for sputum smear, culture and Xpert were calculated. For patients underwent all the three tests, we compared frequency distribution of characteristics between bacteriologically positive patients and negative patients using conventional 2-way contingency tables, tested statistical significance using χ^2^ test. The logistic regression was used to analyze factors associated with BC among pulmonary TB patients underwent all three tests. With BC as the dependent variable in the model, characteristics were introduced as independents one by one, calculating odds ratios (OR) and 95% confidence intervals (CI), and then all characteristics were initially included as independents using backward method to select the final model, calculating adjusted OR (aOR) for all factors. All the analyses were carried out by using SAS 9.4 (SAS Institute, Cary, NC), α = 0.05.

### Ethic review

This study used de-identified data reported to TBIMS through routine patient care-linked surveillance. The protocol was reviewed by review board of Tianjin Center for Tuberculosis Control. All data used for this study obtained permission from the review board. Informed consent for the patients and ethical approval was not required for the study.

## Results

### Patient profile

**Fig. 1:**
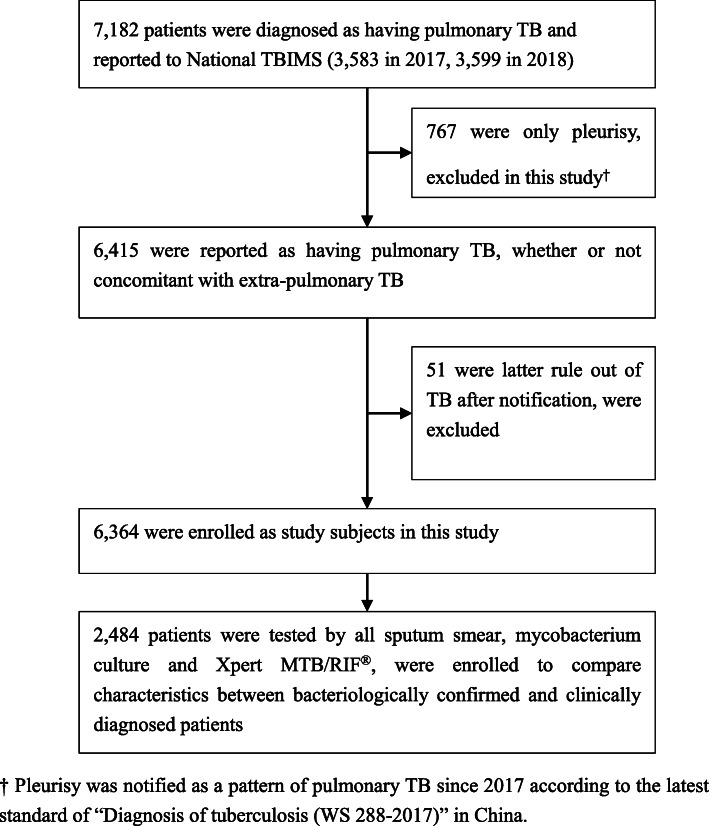
Flow-chart of study subjects recruitment

During 2017 to 2018, a total of 6415 pulmonary TB patients were reported to TBIMS (767 pure pleurisy not included) in Tianjin ((Fig. 1)). Among them, 51 patients were later ruled out of TB, including 28 initially culture positive ones (corrected as *Mycobacterium other than TB*) and 23 initially culture negative ones (corrected as lung cancer, silicosis, pneumonia and so on). The remaining 6364 patients were enrolled as study subjects, including 3159 reported in 2017 and 3205 in 2018. Among them, 4383 (68.9%) were male, 1981 (31.1%) were female. The age ranged between 1 to 95 years (skewness = 0.16, *P* < 0.01); age < 15 years accounted for 30 (0.5%); the median age was 48 years (IQR: 28, 63). Local registered residents accounted for 5626 (88.4%), and migrants accounted for 738 (11.6%). New patients accounted for 5356 (84.2%), and previously treated patients accounted for 1008 (15.8%). At the first arrival in TB facilities, 3079 (48.4%) patients had symptoms ≥2 weeks, and 3285 (51.6%) had symptoms < 2 weeks. Culture was tested for 5853 (92.0%) of the patients on initial sputum, respectively accounting for 90.7% (2864/3159) of patients reported in 2017, and 93.3% (2989/3205) of patients reported in 2018. A total of 2605 (40.9%) patients were tested Xpert on initial sputum, respectively accounting for 2.5% (80/3159) of patients reported in 2017 and 78.8% (2525/3205) of patients reported in 2018.

### Positivity yield by different strategies

A total of 4181 (65.7%) patients were bacteriologically confirmed, including 1953 (61.8%) reported in 2017 and 2228 (69.5%) in 2018, and the rest 2183 (34.3%) were clinically diagnosed (Table 1). When using as a single tool, positivity was 43.2% (2746/6364) for smear microscopy, 57.8% (3380/5851) for culture, and 61.7% (1608/2605) for Xpert. Overall, 2484 (39.0%) of study subjects were tested simultaneously by smear, culture and Xpert on initial sputum, and 73.4% (1824/2484) were positive shown by any of the tests (shown in Table 1). The yields of positivity were statistically different in various combinations of the testing methods (*P* < 0.01). Regardless the testing methods, an add-on could always significantly achieve additional positivity. Adopting culture as an add-on testing to smear, an extra 19.9% positivity was achieved; Xpert as an add-on test to smear, 24.7% extra positivity achieved. When combining the three, yield of positivity was 73.4%, the highest among all the strategies.

**Table 1:** Positivity yield by different bacteriological testing strategies among pulmonary TB patients in Tianjin China, 2017–2018

Testing strategy (n)	Negativity n (%)	Positivity n (%)
Smear (6364)	3618 (56.9)	2746 (43.1)
Culture (5853)	2473 (42.3)	3380 (57.7)
Xpert (2605)	997 (38.3)	1608 (61.7)
Smear+culture (5853)	2162 (36.9)	3691 (63.1)
Smear+Xpert (2605)	838 (32.2)	1767 (67.8)
Xpert+culture (2484)	702 (28.3)	1782 (71.7)
Smear+culture+Xpert (2484)	660 (26.6)	1824 (73.4)

### Comparison between bacteriologically positive and negative patients

Bacteriological testing results were significantly associated with several patient characteristics (*P* < 0.01, shown in Table 2). The positivity rate of older aged (≥45) was higher than that of younger aged (< 45). As age increased, the positivity proportion went up from 63.6% among the < 25 years to 83.3% among the ≥65 years, the trend was statistically significant (*P* < 0.01). In terms of occupation, the un-employed, farmers and retirees had higher positivity rate (> 70%) than that of students, service/manufacture workers and state employees (< 70%). Regarding hospital type of TB diagnosis, the city-level TB designated hospital (with inpatient wards) was over presented among the positive group than TB clinics. Besides, local registered residents compared with migrants, having symptoms ≥2 weeks before arriving in TB health care facilities, previously treated, symptoms with cough or blood sputum, with cavitation on chest X-ray, bilateral lobes of lung affected, and diabetes comorbidity were all significantly over presented among the positive patients than the negative ones. Gender, ethnicity and extra-pulmonary TB concomitance were not significantly associated with BC (*P* > 0.05).

**Table 2:** Comparison of characteristics between bacteriologically positive and negative patients who underwent all sputum smear, culture and Xpert in Tianjin, China, 2017–2018

Characteristics (n)	Negativity (%)	Positivity (%)	Total	P by χ^2^
Gender (2484)				
Male	434 (65.8)	1265 (69.4)	1699	0.09
Female	226 (34.2)	559 (30.6)	785	
Age (2484)				
< 25 yr ^a^	162 (24.5)	283 (15.5)	445	< 0.01
25–44	241 (36.5)	513 (28.1)	754	
45–64	173 (26.2)	608 (33.3)	781	
≥ 65 yr	84 (12.7)	420 (23.0)	504	
Ethnic group (2484)				
Han	649 (98.3)	1788 (98.0)	2437	0.62
Other	11 (1.7)	36 (2.0)	47	
Migrant (2484)				
No	535 (81.1)	1641 (90.0)	2176	< 0.01
Yes	125 (18.9)	183 (10.0)	308	
Occupation (2484)				
Student	99 (15.0)	148 (8.1)	247	< 0.01
Unemployed	107 (16.2)	512 (28.1)	619	
Farmer	68 (10.3)	218 (12.0)	286	
Service/manufacture worker ^b^	75 (11.4)	126 (6.9)	201	
State employee	179 (27.1)	355 (19.5)	534	
Retiree	90 (13.6)	314 (17.2)	404	
Not provided/unclear	42 (6.4)	151 (8.3)	193	
Hospital pattern of diagnosis (2484)				
City-level TB clinic	237 (35.9)	255 (14.0)	492	< 0.01
City-level TB hospital ^c^	286 (43.3)	1337 (73.3)	1623	
District-level TB clinics	137 (20.8)	232 (12.7)	369	
Time for having TB symptoms ^d^ (2,484)				
< 2 weeks	413 (62.6)	809 (44.4)	1222	< 0.01
≥ 2 weeks	247 (37.4)	1015 (55.6)	1262	
Previously treated (2484)				
No	601 (91.1)	1549 (84.9)	2150	< 0.01
Yes	59 (8.9)	275 (15.1)	334	
Symptoms with cough (2463)				
No	412 (62.5)	641 (35.5)	1053	< 0.01
Yes	247 (37.5)	1163 (64.5)	1410	
Symptoms with blood sputum (2463)				
No	618 (93.8)	1598 (88.6)	2216	< 0.01
Yes	41 (6.2)	206 (11.4)	247	
Cavitation on chest X-ray (2445)				
No	574 (87.5)	1099 (61.4)	1673	< 0.01
Yes	82 (12.5)	690 (38.6)	772	
Bilateral lung lobes affected (2445)				
No	435 (66.3)	787 (44.0)	1222	< 0.01
Yes	221 (33.7)	1002 (56.0)	1223	
Diabetes comorbidity (2303)				
No	560 (91.2)	1412 (83.6)	1972	< 0.01
Yes	54 (8.8)	277 (16.4)	331	
Extra-pulmonary TB concomitance (2303)				
No	557 (90.7)	1492 (88.3)	2049	0.11
Yes	57 (9.3)	197 (11.7)	254	

^a^ Totally 30 were age < 15, among whom 20 were bacteriologically negative and 10 were bacteriologically positive. ^b^ Occupations in food industry, public transportation, public service attendants, and factory workers. ^c^ Designated TB hospital with inpatients. ^d^ Defined as the period from symptoms onset to the first arrival in TB facilities

### Factors associated with bacteriological positivity

In logistic regression, gender, age, ethnicity, residency, previous treatment, diabetes comorbidity and extra-pulmonary TB concomitance were not statistically associated with BC (*P* > 0.05, shown in Table 3). The other characteristics were identified to significantly increase or decrease bacteriological positivity. Compared with students, the un-employed and farmers had significant higher risk of being positive. Compared with patients diagnosed in the city-level TB clinic, patients in city level TB designated hospitals (with inpatients) were associated with BC. Patients arrived in TB facilities ≥2 weeks since onset of TB symptoms increased bacteriological positivity compared with those arrived timelier. Symptoms with cough, blood sputum, cavitation on chest X-ray and bilateral lobes of lung affected were all associated with increased BC.

**Table 3:** Factors associated with bacteriological positivity among pulmonary TB patients in Tianjin China, 2017–2018

Characteristic (n)	OR (95% CI)	aOR (95% CI)	*P by* *wald χ2*
Gender (2484)			
Male	ref	ref	0.85
Female	0.8 (0.7–1.0)	1.0 (0.8–1.2)	
Age (2484)			
< 25 yr ^a^	ref	ref	0.19
25–44	1.2 (1.0–1.6)	1.1 (0.7–1.5)	
45–64	2.0 (1.6–2.6)	1.2 (0.8–1.9)	
≥ 65 yr	2.9 (2.1–3.9)	1.6 (1.0–2.7)	
Ethnic group (2484)			
Han	ref	ref	0.66
Minority	1.2 (0.6–2.3)	1.2 (0.5–2.9)	
Migrant (2484)			
No	ref	ref	0.37
Yes	0.5 (0.4–0.6)	1.2 (0.8–1.6)	
Occupation (2484)			
Student	ref	ref	0.02
Unemployed	3.2 (2.3–4.4)	1.5 (1.0–2.2)	
Farmer	2.1 (1.5–3.1)	1.7 (1.1–2.8)	
Service/manufacture ^b^	1.1 (0.8–1.6)	1.2 (0.7–1.9)	
State employee	1.3 (1.0–1.8)	0.9 (0.6–1.4)	
Retiree	2.3 (1.7–3.3)	1.3 (0.8–1.9)	
Not provided/unclear	2.4 (1.6–3.7)	0.9 (0.5–1.5)	
Hospital pattern of diagnosis (2484)			
City-level TB clinic	ref	ref	< 0.01
City-level TB hospital ^c^	4.3 (3.5–5.4)	3.4 (2.6–4.4)	
District-level TB clinics	1.6 (1.2–2.1)	0.8 (0.6–1.2)	
Time for having TB symptoms ^d^ (2,484)			
< 2 weeks	ref	ref	0.00
≥ 2 weeks	2.1 (1.7–2.5)	1.4 (1.1–1.8)	
Previously treated (2484)			
No	ref	ref	0.05
Yes	1.8 (1.3–2.4)	1.4 (1.0–2.0)	
Symptoms with cough (2463)			
No	ref	ref	< 0.01
Yes	3.0 (2.5–3.6)	2.2 (1.8–2.8)	
Symptoms with blood sputum (2463)			
No	ref	ref	0.04
Yes	1.9 (1.4–2.8)	1.5 (1.0–2.2)	
Cavitation on chest X-ray (2445)			
No	ref	ref	< 0.01
Yes	4.4 (3.4–5.6)	3.3 (2.5–4.3)	
Bilateral lung lobes affected (2445)			
No	ref	ref	< 0.01
Yes	2.5 (2.1–3.0)	1.7 (1.4–2.2)	
Diabetes comorbidity (2303)			
No	ref	ref	0.06
Yes	2.0 (1.5–2.8)	1.4 (1.0–2.0)	
Extra-pulmonary TB concomitance (2303)			
No	ref	ref	0.20
Yes	1.3 (0.9–1.8)	1.3 (0.9–1.8)	

^a^ Totally 30 were age < 15, among whom 20 were bacteriologically negative and 10 were bacteriologically positive. ^b^ Occupations in food industry, public transportation, public service attendants, and factory workers. ^c^ Designated TB hospital with inpatients. ^d^ Defined as the period from symptoms onset to the first arrival in TB facilities

## Discussion

In China, decline of BC proportion among pulmonary TB was not only reflected by the surveillance data, but also shown from the latest two national TB surveys respectively conducted in 2000 and 2010, that as the prevalence of active pulmonary TB nearly remained (from 466/100,000 to 459/100,000), whereas prevalence of bacteriologically positive pulmonary TB decreased (from 216/100,000 to 119/100,000), and even more sharply for the smear positive ones (from 169/100,000 to 66/100,000), meaning BC proportion shrank from 46.4 to 25.9%, and smear-positivity proportion from 36.3 to 14.4% [11]. Similar trend was reported by some other regional data, that as smear-negative TB incidence remained, the incidence of smear-positive TB declined [12]. Globally, BC proportion declined from 56% in 2017 to 55% in 2018 [1, 13]. Given testing methods are on evolution, MTB testing strategy should be improved to keep in pace with the decline of BC.

In our study, around 2/3 the notified pulmonary TB were bacteriologically confirmed, higher than the average level of the world, and even higher than the national level. This may be explained as follows. One was integration of sputum tests for pulmonary TB patients, which was shown by the fact that as coverage of culture (from 90.7 to 93.3%) and Xpert (from 2.5 to 78.8%) increased during the two years, the BC proportion improved significantly (from 61.8 to 69.5%). Xpert being used as an initial diagnostic test in all adult/children presumable patients has been recommended in WHO guidelines [9]. Either culture or Xpert had higher positivity yield than smear when using alone, so both culture and Xpert can be adopted as add-on tools to maximize BC. The second point was the diagnostic procedure of pulmonary TB in the absence of bacteriological evidence, that a specific panel of physicians rather than a individual physician was required to make the diagnosis to minimize over-diagnosis or misdiagnosis [6]. Misclassification of people as active TB patients will not only enlarge denominator for BC proportion, but also more seriously do harm to the patients. The third point was a regional feature in the city, that over 80% of the patients were diagnosed in two city-level designated TB facilities, where the TB labs receive direct quality control by the national TB reference lab. Potent quality control for a TB lab is essential for successful detection of MTB from sputum specimens [14]. The high BC proportion in Tianjin has set an example to determine what level can be achieved in the whole country and other regions with the similar problem. Implementation of procedure for TB diagnosis in the absence of bacteriological evidence, good quality control for TB labs, and combination of sputum tests could be considered as measures to improve BC of pulmonary TB patients.

However, the BC proportion in Tianjin was still lower than that of some regions. In the USA 78.1% of TB cases were confirmed via culture and an extra 2.9% were confirmed through positive nucleic acid amplification [15]. In New York 85% of the pulmonary TB patients were culture positive [16]. The gap may reflect different background of TB prevalence in countries/regions, and also partly attribute to different profile of patient characteristics. Apart from factors relating to laboratory, patients with specific characteristics tend to have pauci-bacillary, such as HIV co-infection, children, and mild clinical manifestations [17–19].

Generally, more severe and complex condition tend to cause bacteriological positivity. Cavitation on chest X-ray usually means enriched bacteria in the lesion and represents later stage of the disease, and cough can produce sputum containing MTB from the lesion to the air through droplets, increasing the possibility to detect MTB in sputum samples, which is in consistent with previous studies [4, 16, 19, 20]. Similarly, blood sputum and bilateral lung affected may reflect extensive lesion, which were also found to increase BC proportion. Diabetes comorbidity were found to be associated with higher grading of sputum smear among TB patients in other studies [7, 21, 22]. However in this study, the effect of diabetes to BC was marginal, probably because this condition had been represented by comprehensive severity and complexity of other factors. Patients with complex conditions in TB or comorbidities tended to be referred to and admitted by health care facilities with inpatient wards rather than TB clinic. And this selection bias might explain that in this study diagnosis in city level hospital with inpatient wards was associated with higher BC proportion. Apart from clinic manifestation, seeking TB care after prolonged symptoms tend to cause bacteriological positivity in this study. In previous studies, treatment delay was found to be a risk factor associated with TB transmission from index patients to the contacts, although different cut-off points for categorizing treatment delay were used [4, 23]. Prolonged symptoms mean longer period for the deterioration of the disease, and also make contacts longer time under exposure of transmission.

Regarding demographic factors, gender and age were found related to bacteriological results in some studies [16, 19]. In our study, although the older age were over presented in the bacteriologically confirmed group, after adjusted with other factors, age as well as gender were not statistically significant. Compared with state employees, the unemployed and farmers usually had disadvantage in household income and living conditions, leading them vulnerable to TB [24, 25]. The vulnerability might lower their health seeking behavior or accessibility to health care, thus lead to later stage of the disease. As TB control program being enhanced and active TB case finding being carried out to larger population, less patients may be diagnosed at later and severe stage of the disease, as a result BC may decrease if testing strategy remain. Intervention on sputum collection such as induced sputum using nebulization can be considered as an option to improve BC among presumable TB patients who tend to be bacteriologically negative [26]. However, diagnosis of pulmonary TB should not simply relay on BC, which may cause delay for anti-TB treatment [5]. For patients tend to be bacteriological negative, physicians should still keep vigiliant on TB diagnosis before bacteriological results, take into account of comprehensive conditions such as chest X-ray of the patients and opinions from a diagnosis panel, to minimize delay for treatment [2, 6].

Limitation of the study: the study was based on surveillance data reported to TBIMS, and history of broad spectrum anti-bacterium medication before TB diagnosis was not routinely reported. For example, Levofloxacin is broadly used in general hospital, which may rapidly reduce the number of bacilli expelled. HIV was found to be associated with infectiousness of TB patients, [4] however in our dataset very low proportion of HIV comorbidity was reported to satisfy the statistics analysis. Despite the limitations, when China and some parts of the world facing the challenge of low BC among pulmonary TB patients, this study revealed an improved proportion achieved regionally, which could be an example to the whole country and some other regions with the similar problem.

## Conclusions

Being unemployed, farmers, having prolonged symptoms, and severity in TB condition were factors associated with BC among pulmonary TB patients. Combination of tests was an effective way to improve BC among pulmonary TB patients. We recommend combination of tests to improve BC for pulmonary TB patients according to regional resource, especially who are in early stage of the disease or with conditions tend to be bacteriologically negative.

## Data Availability

The datasets analyzed during the current study were exported from China National TB Information Management System (TBIMS), excluding identified information. The dataset are available from the corresponding author on reasonable request.

## Abbreviations

TB: Tuberculosis
Xpert: Xpert® MTB/RIF assay
BC: bacteriological confirmation
MTB: *Mycobacterium tuberculosis*
TBIMS: TB Information Management System
